# Impact of the COVID-19 pandemic on the mental and physical wellbeing of patients with motor neuron disease and other neuromuscular disease

**DOI:** 10.3389/fneur.2025.1514983

**Published:** 2025-02-19

**Authors:** Srestha Mazumder, Antonia S. Carroll, Hannah C. Timmins, Matthew C. Kiernan, Colin J. Mahoney

**Affiliations:** ^1^Neuroscience Research Australia, Sydney, NSW, Australia; ^2^Brain and Mind Centre, University of Sydney, Sydney, NSW, Australia; ^3^School of Clinical Medicine, University of New South Wales, Sydney, NSW, Australia; ^4^Department of Neurology, Prince of Wales Hospital, Sydney, NSW, Australia; ^5^South Western Sydney Local Health District, Sydney, NSW, Australia

**Keywords:** motor neuron disease, chronic inflammatory demyelinating polyneuropathy, multifocal motor neuropathy, COVID-19, physical well-being, mental health

## Abstract

**Background and aims:**

During the COVID-19 pandemic, vulnerable populations faced worsening mental and physical well-being due to limited access to support systems and diverted health resources during lockdowns. Individuals with chronic neurological disorders including motor neuron disease (MND), chronic inflammatory demyelinating polyneuropathy (CIDP), and multifocal motor neuropathy (MMN) were at considerable risk of severe COVID-19 symptoms. The present study aimed to examine the psychological and physical impact of lockdowns on individuals with MND and other chronic neuromuscular disorders (non-MND).

**Methods:**

Online surveys were distributed to 58 patients, with information prospectively collected to capture demographics, COVID-19 concerns, resilience, loneliness, anxiety, and depression using validated measures. Disease severity and physical activity levels were also assessed. Data was analysed using Mann–Whitney U and Chi-square tests.

**Results:**

MND patients consistently showed resilience regardless of their impairment level. In further support, those with non-MND conditions reported greater concern for their mental well-being and experienced significantly more loneliness than MND patients (*p* = 0.005). Moderately to highly impaired non-MND patients experienced higher levels of loneliness (*p* = 0.024), anxiety (*p* = 0.006), and depression (*p* < 0.001) compared to similarly impaired MND patients.

**Conclusion:**

These results suggest that despite having a poorer prognosis, MND patients demonstrate resilience, possibly reflecting increased social and allied health support. Neurobehavioral differences may also contribute to differing illness beliefs and behaviours. In the event of future pandemic events, additional targeted social supports, recreational activities, and allied health interventions may have a greater impact in reducing distress for those with CIDP and MMN.

## 1 Introduction

In the wake of the coronavirus disease (COVID-19) global health crisis, stringent public health and social measures at individual and community levels were imposed in an attempt to control the spread of infections. These measures included public education, travel restrictions, closure of non-essential services and physical distancing, and were aptly termed ‘lockdown’. In late June of 2021, New South Wales (NSW), Australia was subject to stringent lockdowns resulting from the Delta variant of COVID-19 with restrictions on travel and participation in outdoor exercise. This lockdown continued until October 2021, the longest recorded lockdown for NSW.

A key goal of the lockdown was to keep the most vulnerable individuals safe from infection. This group of people were characterised as individuals over the age of 70, immunocompromised states and those with existing medical, psychiatric or substance abuse problems (1). However, this classification did not identify patients with specific diseases. A meta-analysis suggested that those with pre-existing neurological diseases had a 47% greater chance of dying from COVID-19, highlighting that the presence of neurological diseases increased risk beyond that conveyed by general COVID-19 risk factors (2, 3). Furthermore, patients with respiratory insufficiency due to neuromuscular weakness such as motor neuron disease (MND) (4, 5) and rare chronic inflammatory demyelinating polyneuropathy (CIDP) (6, 7), were more likely to develop severe illness from COVID-19 and be subject to worse outcomes including requirement of and dependence on assisted ventilation.

COVID-19 safe practices, including social distancing and isolation were recommended to reduce the risk of COVID-19 transmission, however they also presented potential consequences on the physical and mental well-being of these at risk patient groups. Specifically, social distancing and strict lockdown measures likely impacted the ability of this population to maintain physical activity and structured exercise, thereby affecting muscle strength, respiratory function, fatigue, and overall motor function (8, 9). Research has shown that monitored exercise programmes can help reduce motor deterioration in MND patients, decreasing complications from muscle atrophy and allowing maintenance of mobility for longer (10). Similar impacts are observed in those with peripheral neuropathy where exercise is used as a form of treatment for disease related fatigue, and to improve fitness, function and muscle strength (11, 12).

During lockdown, peak medical bodies launched nationwide campaigns to encourage people to prioritise their health during the pandemic, as it became apparent that loneliness and physical activity were critical mediators of mental health (13). Early studies during the pandemic linked loneliness with worsening mental health and heightened psychological distress, particularly in vulnerable populations (14); especially for individuals who engaged in minimal activity and social interactions (15). For example, patients with neuromuscular disease were expected to experience poorer mental health due to isolation from family, lockdown measures, fear of contracting COVID-19, and a lack of access to healthcare during that time (16–18). This combination of unwanted mental and physical changes may have served to complicate clinical care and potentially accelerated disease progression well after the lockdowns ended (19, 20).

As such, the current study analysed the psychological and physical effects of the lockdowns in NSW. Our aims were to establish how lockdowns impacted mental health (as measured by levels of anxiety, depression, loneliness, resilience) and physical health (as measured by activity and exercise) in those with MND and other non-MND neuromuscular disorders (CIDP and MMN). Based on established disease trajectories we hypothesised that (i) MND patients would exhibit more mental health problems compared to non-MND patients diagnosed with a chronic neuromuscular condition, and that (ii) non-MND patients may exhibit more resilience and involvement with exercise and activity.

## 2 Materials and methods

Suitable patients were identified from a research database located at a tertiary neuromuscular disorders clinic in Sydney, Australia from 1st January 2020 to10th May 2022. This timeframe was chosen to include all patients who were followed up clinically during the COVID-19 lockdowns, noting the NSW lockdown for civilians commenced on 31st March 2020 and ended on 11th October 2021 with intermittent periods of respite where harsh restrictions were briefly lifted or minimised. Participants were recruited prospectively and were included only if they underwent clinical assessment during the period of lockdown. Two authors (CJM and SM) reviewed each participant’s clinical data to confirm the diagnosis of MND, CIDP or MMN met published criteria (21–23). Patients were categorised into two groups: MND and non-MND, the latter group included patients with CIDP and MMN. Those with uncertain diagnoses were excluded from the study.

Patients were provided with an electronic link to a RedCap survey specific to their respective disease group. The survey consisted of two parts. The first part was a questionnaire designed by the authors to collect patient demographics, their experiences with mental health, COVID-19 vaccination status, and coping mechanisms during the pandemic lockdown in NSW (see Supplementary material). The second part comprised of a compilation of previously published questionnaires assessing disease-specific functional impairment, mental and physical health (refer to Table 1). The chosen functionality questionnaires prioritise lower limb parameters to evaluate the patient’s ability to engage in exercise and activity. Patients provided consent for the study by returning the survey. A total of 30 MND and 28 non-MND patients responded to the survey. This study was approved by the University of Sydney’s HREC (2020/ETH01090).

**Table 1:** Questionnaires provided patients respective to their disease group.

MND	NON-MND	Assessment
Demographic questionnaire	Demographic questionnaire	7-item questionnaire gathering participant gender, date of birth, education, marital status, employment status and occupation data.
Experience with COVID-19 and mental health questionnaire	Experience with COVID-19 and mental health questionnaire	7-item questionnaire gathering data on participant experience with COVID-19 and the vaccine.
Amyotrophic Lateral Sclerosis Functional Rating Scale - Revised (ALSFRS-R)	Inflammatory Rasch-built Overall Disability Scale (I-RODS)	**ALSFRS:** 12-item assessment of functionality and activity across 4 domains (Bulbar, Fine Motor, Gross Motor and Respiratory), scored out of 48. Lower scores indicate more impairment (51). **I-RODS:** 24-item assessment of activity and social participation for immune mediated peripheral neuropathies, scored out of 48. Lower scores indicate more impairment (52).
UCLA Loneliness Scale Version 3	UCLA Loneliness Scale Version 3	20-item measure assessing feelings of disconnection from others. Scored out of 80, where higher scores indicate more loneliness (53).
Hospital Anxiety Depression Scale	Hospital Anxiety Depression Scale	14-item questionnaire (7 for anxiety, 7 for depression) measuring anxiety and depression. The scale is scored separately where scores below 7 indicate non-cases. Higher scores indicate more severe symptoms (54).
Resilience Scale for Adults	Resilience Scale for Adults	33-item questionnaire scored out of 231 on a Likert scale ranging from 1 to 7. Higher scores indicate more resilience (55).
Veteran Specific Activity Questionnaire	Veteran Specific Activity Questionnaire	13-item self-reported symptom questionnaire measuring aerobic fitness and exercise tolerance. Activities are listed in increasing difficulty and individuals choose one item from list that causes them cause discomfort when completed for a period of time (56).
Incidental and planned exercise questionnaire	Incidental and planned exercise Questionnaire	12-item measure assessing frequency and duration of planned and incidental physical activity. Higher durations indicate more time spent engaging in planned and incidental physical activity (0 to 182 h) (57).

Data were analysed using Jamovi statistical software version 2.3.26, a graphical interface for the R programming language. Descriptive statistics were calculated to summarise the data. Mann–Whitney U tests were performed to assess differences between the two disease groups across the questionnaires.

To assess differences in mental and physical well-being based on disease severity, patients from each disease cohort were categorised into two severity groups using disease-specific questionnaires: ALSFRS-R for MND and I-RODS for non-MND. Mann–Whitney U tests were performed to examine differences between and within disease groups concerning disease severity.

The ALSFRS-R is a 12-item assessment with each item scored on a scale of 0 to 4. A score of 4 is unrestricted functionality and 0 is complete loss of functionality and dependence. Based on previously published cut-offs for severity we categorised patients into those with no or mild impairment (≥35/48) and those with moderate to high impairment (<35/48) (24).

The I-RODS is a 24-item assessment scored on a scale of 0 to 2 where a score of 2 indicates the patient can easily perform a task, 1 indicates difficulty performing tasks, and 0 indicating a task is impossible to perform. Based on the dissection of these scores, patients who scored 35 to 48 were categorised as having low impairment and those scoring below 34 were categorised as having moderate to high impairment. This delineation is to reflect the individuals who provide mixed scores of 2 and 1 indicating mild impairment (25).

## 3 Results

In total, 30 MND and 28 non-MND patients participated in the study with an almost equal number of female and male respondents (see Table 2). There was a significant difference in age (*p* < 0.05; see Table 1) where MND patients were older on average than non-MND patients. Additionally, the two groups differed significantly in their disease duration, (*p* < 0.001; see Table 2), with non-MND patients having had their disease for a longer period. This was expected due to the fast-progressing, terminal nature of MND and the average age of onset being later in life. The majority of patients in both groups had been COVID-19 vaccinated; 90% of MND patients and 71% non-MND.

**Table 2:** Demographics table.

	MND	Non-MND	*p*
Number of patients (M:F)	30 (15:15)	28 (15:13)	–
Age (years) ± SD	66.7 ± 8.28	58.3 ± 13.9	*p* < 0.05
Disease duration (months) between diagnosis and baseline assessment ± SD	65.8 ± 111	117 ± 71.9	*p* < 0.001
Lower Limb onset %	43%	53%	–
Covid-19 vaccinated %	90%	71%	–
ALSFRS-R (average) ± SD	33 ± 9.58	n/a	–
I-RODS (average) ± SD	n/a	37.6 ± 8.40	–
Non-invasive ventiliation	23%	n/a	–
Gastronomy use	13%	n/a	–
IVIG use	n/a	39%	–
Corticosteroids	n/a	7%	–

**Figure 1:**
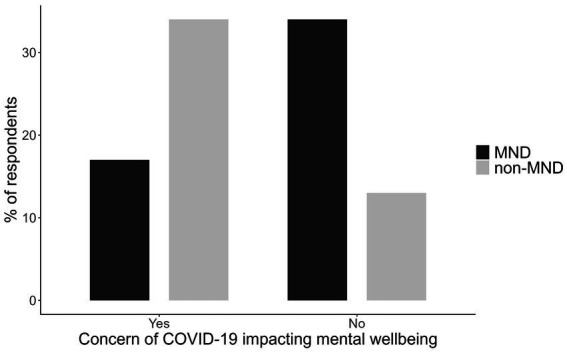
The proportion of MND and non-MND patients respondents concerned about the impact of COVID-19 on their mental wellbeing.

Patients were asked if they were concerned about the COVID-19 pandemic impacting their mental wellbeing. A chi-square analysis was conducted to examine the differences in reported concern. The results revealed a significant association, χ^2^(1, N = 58) = 8.42, *p* = 0.004, indicating that the level of concern differed significantly between the two groups. Non-MND patients expressed having more concerns of the COVID-19 pandemic on their mental health than MND patients (Figure 1).

### 3.1 Mental and physical health stratified by disease

A Mann–Whitney U test was used to determine if MND and non-MND patients differed in their experiences of mental and physical wellbeing (see Tables 3, 4). With regards to mental health the most significant difference was observed on a measure of loneliness (U = (221), *p* = 0.005, 95% CI [−16.0, −3.0]), where non-MND patients (mean = 49.04) expressed more feelings of loneliness compared to MND patients (mean = 39.38). This was followed by anxiety (U = (264), *p* = 0.054, 95% CI [−4.0, 0.001]) where non-MND patients showed higher levels of anxiety (mean = 6.63) compared to MND patients (mean = 4.68). There was no significant difference between the MND and non-MND groups on measures of depression or resilience.

**Table 3:** Descriptive statistics for psychological measures.

Disease group	Assessment	Mean	Std. dev	Min	Max	*p*
MND	Loneliness	39.38	10.58	18	61	<0.005
Non-MND	Loneliness	49.04	12.52	29	81	
MND	Anxiety	4.68	3.49	0	14	=0.054
Non-MND	Anxiety	6.63	4.16	0	18	
MND	Depression	5.61	3.27	1	12	=0.986
Non-MND	Depression	5.96	4.46	0	17	
MND	Resilience	182.56	24.59	127	229	=0.376
Non-MND	Resilience	172.96	37.02	65	223	

**Table 4:** Descriptive statics for physical measures.

Disease group	Assessment	Mean	Std. dev	Min	Max	*p*
MND	VSAQ	2.86	1.83	1	9	<0.001
Non-MND	VSAQ	4.70	2.35	1	10	
MND	Total activity	13.71	13.7	0	48.3	<0.001
Non-MND	Total activity	33.62	21.5	0.875	80.5	

With regards to estimates of physical activity earlier physical exhaustion and fatigue, as measured by the VSAQ (the values from which are equivalent to the metabolic equivalent of a task), were significantly more likely (U = (194), *p* = 0.001, 95% CI [−3.00, −1.00]) in those with MND (mean = 2.86) compared to non-MND individuals (mean = 4.70). Levels of total physical activity were significantly lower (U = (161), *p* < 0.001, 95% CI [−26.88, −9.125]) in MND patients (mean = 13.71) compared to non-MND individuals (mean = 33.62).

### 3.2 Mental and physical health stratified by disease severity

To determine if disease severity impacted mental and physical wellbeing, we stratified each group by their level of disease severity. A within-group Mann–Whitney U test was conducted to compare mental and physical wellbeing for MND and non-MND participants. For the MND cohort disease severity was graded as mild or moderate to high, based on individual scores measured by the ALSFRS-R. Disease severity did not influence mental or physical health in MND participants across any measures (not significant).

For the non-MND cohort disease severity was graded as mild or moderate to high based on their individual scores as measured by the I-RODS. Those with moderate to high disease severity reported significantly more depression (mean = 10.10 vs. 3.533, U = 12.50, *p* < 0.001, 95% CI [−9.00, −4.00]), anxiety (mean = 9.60 vs. 4.88, U = 30.50, *p* = 0.006, 95% CI [−8.00, −2.00]), and loneliness (mean = 56.50 vs. 44.65, U = 39.50, *p* = 0.024, 95% CI [−21.00, −1.00]) compared to the low impairment group. The two groups did not differ in their resilience (U = 42.50, *p* = 0.051, 95% CI [−0.001, 60.00]), but approached significance. In terms of physical activity, the low impairment group completed significantly more activity (mean = 40.60 vs. 21.74, U = 37.00, *p* = 0.0015, 95% CI [3.19, 36.75]) than the moderate impairment group. The moderate to high impairment group was found to report exertion at significantly lower thresholds (mean 2.60 vs. 5.94, U = 8.50, *p* < 0.011, 95% CI [2.00, 4.00]) compared to the low impairment group.

### 3.3 Impact of disease severity: between group comparison

To examine how disease severity impacted physical and mental health differently across MND and non-MND patients, a between-group Mann–Whitney U test was conducted, Non-MND patients graded as moderate to high disease severity reported significantly higher levels of loneliness (mean = 56.50 vs. 39.93, U = 22.5, *p* = 0.006, 95% CI [−26.00, −4.00]), anxiety (mean = 9.60 vs. 5.15, U = 27.0, *p* = 0.019, 95% CI [−8.00, −1.00]) and depression (mean = 10.10 vs. 6.38, U = 30.5, *p* = 0.034, 95% CI [−7.00, −0.001]) compared to the MND group with the same level of impairment. However, there was no significant difference in resilience between the groups. In terms of physical activity, both disease groups engaged in similar amounts of total activity and reported similar levels of exertion during activity (not significant).

Even when stratified into the low impairment group, MND and non-MND patients exhibited significant differences in their physical wellbeing. Non-MND patients completed more total activity (mean = 40.60 vs. 16.32, U = 38.5, *p* < 0.001, 95% CI [−38.750, −9.00]) compared to MND patients. Additionally, MND patients reported fatigue at lower thresholds compared to non-MND patients (mean = 3.21 vs. 5.94, U = 31.0, *p* < 0.001, 95% CI [−4.00, −1.00]). In regards to mental wellbeing there were no significant differences between the two disease groups on any of the measures (*not significant*).

## 4 Discussion

The present study examined the effect of the COVID-19 lockdowns on the mental and physical wellbeing of patients across the spectrum of neuromuscular disease. Based on the terminal nature of MND, we anticipated MND participants to report higher burdens of mental and physical health (26). Instead, we found that non-MND patients expressed greater concerns about the potential health impact of COVID-19 compared to those with MND.

CIDP and MMN are not generally considered to be terminal disorders, and many are able to maintain functionality in the workplace, home and socially. Our data suggests that the pandemic disproportionally increased symptom burden in this population. We speculate that this group, with a more chronic disease pathology, may have experienced a greater disruption to normal routines aimed at maintaining wellbeing compared to those dealing with more acute and aggressive disease. Perceptions of harm in this group may be heightened due to COVID-19’s reported association with worsening neuropathies like CIDP and Guillain-Barré syndrome (27). Furthermore, these individuals may have greater perceived risk of COVID-19 infection in the setting of immunosuppressive therapies and potential for attenuated vaccination responses, thereby compounding anxieties (18). Of note in the current study, the non-MND cohort exhibited lower COVID-19 vaccination rates compared to the MND cohort, possibly due to perceived increased risk of GBS or exacerbation of inflammatory neuropathies (28). This reduced vaccination uptake could also be associated with decreased engagement in social and physical activities, leading to increased feelings of loneliness.

Comparatively, lower levels of mental health concern were seen in those with MND. This could be due to differences in disease appraisal, as most MND patients recognise they have a terminal illness, which they may have viewed as more serious than COVID-19, and as such they continued to prioritise maintaining mental wellbeing. Particularly, MND patients utilise coping strategies such as acceptance, and seeking support from family and friends and positive reinterpretation of their situation which promotes resilience (29). Indeed, studies have shown that traits such as resilience are not purely innate but develop throughout life as individuals are exposed to adversity and through external factors such as social and family support (30). Previous studies support our findings and have shown that regardless of MND disease stage and functional impairment, patients reported lower levels of concern over contracting COVID-19 (31). An alternative explanation could relate to underlying neurocognitive changes in those with MND compared with other neuromuscular disorders, where high levels of apathy are common in turn modulating anxiety about other health concerns (32).

Turning to specific themes of mental health impact during lockdown, the non-MND group expressed significant levels of loneliness and anxiety during the pandemic, while levels of resilience and depression were similar, this posits that certain aspects of mental health are more vulnerable than others during periods of lockdown, and in particular isolation. It is noteworthy that those with lower levels of physical activity (i.e., those with MND) had lower levels of loneliness which may result from high dependency on carers for daily activities. Loneliness stemming from isolation from wider family units, friends and colleagues was felt more significantly for the non-MND groups likely due to the sudden and drastic changes to their lifestyle. Conversely those with MND may have had the opportunity to spend greater time at home with loved ones which they appreciated given the terminal nature of their disease. A Japanese study during the pandemic found that those who spent more time with family, were less lonely (33). For this terminally ill population, where illness is more conspicuous to others, it is likely family members made additional efforts to spend time with loved ones acting as an important countermeasure to loneliness. This is an important consideration as loneliness has been linked with worse mental health (34), noting that low social support is a possible proxy for loneliness that can contribute to psychological distress during pandemics.

From a medical care perspective, we would hypothesise that non-terminal neuromuscular diseases patients were not able to access medical support networks to the same extent as patients with MND, which likely increased their anxiety regarding their disease progression. Non-drug costs associated with MND are approximately twice those of CIDP ($61,000 vs. $25,000), which suggests that those with MND have access to better allied health support and/or equipment thereby improving quality of life, which better offsets the mental and physical challenges in times of greater challenge (35). MND patients are linked with a wide network of multidisciplinary services and receive holistic care that may provide them mental and physical resources to navigate challenging situations, such as the lockdown (35, 36) Indeed, this has been observed during COVID-19 where in some regions MND patients were still able to access clinicians and allied health services (37, 38) while CIDP patients had less access to neurology appointments (6). Overall, the lack of social support from colleagues, friends and interactions in the community alongside economic inequities could be a driving factor for the significant levels of loneliness and higher anxiety experienced by non-MND patients (13). Our data suggests that resilience levels were high in both disease groups with multiple studies suggesting this is a fairly crystalised mental resource, which is less vulnerable to ad-hoc events such as a pandemics (39). Conformingly, previous studies have reported stability (40, 41) and constancy in resilience during the COVID-19 pandemic (42). The finding of high levels of resilience in both disease groups and across disease severity further supports this notion. These higher levels of resilience may have also contributed to the finding that levels of depression did not differ significantly when comparing groups (41). Recently, a study found MND patients show greater resilience compared to healthy individuals which also predicted milder mood symptoms such as depression (29). Resilience is seen as a moderator to depression and can help explain why both MND and non-MND patients that expressed high levels of resilience also showed no significant changes in their levels of depression.

### 4.1 Impact of disease severity on mental and physical wellbeing

Previous reports suggest that the primary coping strategy MND patients utilise is acceptance and reframing of their situation (43, 44). Perhaps this is why disease severity in MND patients did not significantly affect their mental or physical health with both groups reporting comparable levels of anxiety, depression, loneliness, resilience and engagement in exercise. Interestingly, both low impairment and high impairment MND patients reported completing comparable amounts of activity and exercise. This was surprising given moderately to highly impaired patients are typically wheelchair bound or have significant trouble mobilising. This may reflect that the questionnaires provide quantitation of perceived physical activities, and that an individual’s perception of their capabilities may differ from reality, with insight perhaps less preserved in those with more advanced MND. Other lines of evidence suggest that patients may overestimate what they are still able to do which could be a sign of cognitive impairment or could be consistent with the “well-being paradox” (45, 46).

Unlike the MND patients, the non-MND patients differed in their mental and physical wellbeing based on disease severity. Non-MND patients with moderate to high levels of impairment were lonelier, more anxious, and depressed. Particularity, their scores of anxiety and depression were in the borderline abnormal ranges. Previous studies have shown MMN and CIDP patients experience more anxiety than healthy controls (47). However, across two measures (HADS and PHQ-2) very few patients with CIDP, MMN and GBS met criteria for depression (18, 47). In addition to increased loneliness, the current group participated in significantly less physical activity, which included both incidental and planned activity. In individuals with more severe disease, there may be a greater interaction between decreased physical activity and loneliness, with the latter identified as a risk factor for deteriorating mental health during the pandemic (13). This could lead to greater feelings of frustration, helplessness, decreased self-esteem, and decreased functional capabilities potentially contributing to heightened anxiety and depression as they become more aware of their limitations (13). Physical limitations may have also limited patients’ abilities to participate in the limited social gatherings allowed during this time, further exacerbating feelings of loneliness.

### 4.2 Limitations

While the average age of our patients was >60 which could have led to the lower response rate as some of the MND and non-MND patients could not have been technologically capable enough to use the online survey Previous studies looking at MND patients during the pandemic have reported findings with similar sample sizes (17, 18). Unfortunately, as this study was completed during the lockdowns, we were not able to facilitate in person data collection. A further limitation is the lack of prospective data on physical and mental wellbeing collected during periods prior to the COVID-19 pandemic, which would have allowed us to more accurately quantify the impact of a global health emergency.

## 5 Conclusion

The study emphasises the vulnerability of mental and physical wellbeing across the spectrum of neuromuscular disease during periods of heightened global stress. It suggests that during these periods illnesses traditionally seen as more serious may actually be better managed than thought and conversely those with more chronic neurological illness are more likely to experience greater challenges to their wellbeing. In some groups the degree of clinical severity may act to predict those that are most vulnerable. Some degree of vulnerability could be predicted based on clinical severity in those with CIDP and MMN. By exploring across a number of physical and mental health domains we have aimed to provide greater granularity on the reasons for impaired wellbeing during a period of heighted global health awareness. In particular we have found that those living with neuromuscular disease reported high levels of resilience, but despite this some experienced disproportionately greater loneliness. This indicates that mental well-being during the pandemic was influenced more by external factors than by the disease itself or the level of impairment.

With future pandemics certain and other global health emergencies increasingly likely, this data suggests that a bespoke approach is essential for maintaining wellbeing during these periods. This, in particular, should focus on identifying those at highest risk, enhancing and maintaining resilience, promoting physical activity, and mitigating anxiety, depression, and loneliness. Increasing community engagement, especially through peer-support groups that employ emotional, informational, and appraisal-based approaches, has been shown to significantly improve quality of life (48, 49). Studies have shown that peer-support programmes developed to address loneliness and isolation were successful in reducing such feelings (50). In the future, models of care such as these should be developed with patients and caregivers, which can be easily enacted during periods of non-standard care.

## Data Availability

The raw data supporting the conclusions of this article will be made available by the authors, without undue reservation.
