# Supplementary material for: Impact of the COVID-19 pandemic on the mental and physical wellbeing of patients with motor neuron disease and other neuromuscular disease
Source: Front Neurol. 2025 Feb 19;16:1514983. doi: 10.3389/fneur.2025.1514983 (PMC11879790; doi:10.3389/fneur.2025.1514983)
Supplement: Supplementary file 1 [file Data_Sheet_1.pdf]

Personal Information

Please enter some information about you

Your Progress

.

Date of birth

____

Gender

◯ Female
◯ Male
◯ Non-binary

E-mail address

____

Education

◯ Primary school (Kindergarten to Year 6 or 7)
◯ Secondary school (Years 7 to 10 or 8 to 10)
◯ Senior secondary school (Years 11 and 12)
◯ Tertiary (University)
◯ Technical or further education institution (e.g., TAFE certificates, diplomas etc.)

Marital Status

◯ Married/De facto
◯ Single/Never Married
◯ Divorced
◯ Seperated
◯ Prefer not to answer

Employment status

◯ Full-time
◯ Part-time
◯ Casual
◯ Away from work
◯ Unemployed
◯ Retired

Occupation (select the one closest to your current/longest employment)

◯ Professionals
◯ Clerical and administrative workers
◯ Technicians and Trades Workers
◯ Managers
◯ Community and personal service workers
◯ Labourers
◯ Sales workers
◯ Machinery operators and drivers

Which of the following experiences of COVID-19, if any, apply to you?

◯ I have tested positive for coronavirus (COVID-19)
◯ I have tested negative for coronavirus (COVID-19)
◯ I suspect I have had coronavirus (COVID-19) based on symptoms, but have not been tested
◯ I currently live with someone with suspected or diagnosed coronavirus (COVID-19)
◯ I am caring for someone with suspected or diagnosed coronavirus (COVID-19)
◯ None of the above
◯ Prefer not to say

Powered by REDCap

Which of the following experiences regarding COVID-19 Vaccination bests describes you?	◯ I have received at least 1 dose of a COVID-19 vaccine<br>◯ I have a confirmed appointment for my first dose of a COVID-19 vaccine<br>◯ I plan to get a COVID-19 vaccination as soon as I can<br>◯ I have decided not to have a COVID-19 vaccine<br>◯ I am undecided about getting a COVID-19 vaccination<br>◯ None of the above
How much has my neurological condition contributed to my decision on a COVID-19 vaccine (0=not at all, 10=very significant)?	0 5 10<br>*(Place a mark on the scale above)*
Which of the following apply to you in regards to mental health? (could tick more than one)	◯ I have lived experience of a mental health issue<br>◯ I support or care for someone with experience of a mental health issue<br>◯ I am a member of the public with an interest in mental health<br>◯ None of the above<br>◯ Prefer not to say
Thinking about the current coronavirus (COVID-19) pandemic, what, if any, concerns do you have about the impact on your mental wellbeing?	__________________________________
We are interested in understanding what people are doing to support their mental wellbeing during the coronavirus (COVID- 19) pandemic. What, if anything, has been helping your mental wellbeing at this time?	__________________________________
